# Utilizing library repository for sexual harassment study in Indonesia: A systematic literature review

**DOI:** 10.1016/j.heliyon.2022.e10194

**Published:** 2022-08-15

**Authors:** Siti Nurbayani, Moh. Dede, Millary Agung Widiawaty

**Affiliations:** a Faculty of Social Sciences Education (FPIPS), Universitas Pendidikan Indonesia, Jln. Dr. Setiabudhi No. 229, Bandung City, West Java, 40154, Indonesia; b Doctoral Program on Environmental Science, Postgraduate School (SPs), Universitas Padjadjaran, Jln. Dipatiukur No. 05, Bandung City, West Java, 40132, Indonesia; c Center for Environment and Sustainability Science (CESS), Universitas Padjadjaran, Jln. Sekeloa Selatan No. 01, Bandung City, West Java, 40132, Indonesia; d National Research and Innovation Agency of Indonesia (BRIN), Jln. MH Thamrin No. 08, Jakarta Pusat, DKI Jakarta, 10340, Indonesia; e Cakrabuana Institute for Geoinformation, Environment and Social Studies (CIGESS), Ciledug, Cirebon Regency, West Java, 45188, Indonesia

**Keywords:** Bachelor's theses, Islamic State University, Open-access, Sexual harassment

## Abstract

Sexual harassment is a social problem that needs urgent attention to reduce its frequent occurrence. This paper is a systematic literature review (SLR) that summarizes the previous studies of sexual harassment from Islamic state universities (UIN) in Indonesia. Data were collected from the final year bachelor's theses available in the online library repository of UIN Syarif Hidayatullah Jakarta, UIN Walisongo Semarang, UIN Sunan Ampel Surabaya, UIN Ar-Raniry Aceh, UIN Alauddin Makassar and UIN Raden Fatah Palembang with open-access facilities. The SLR and Meta-analysis were used to investigate the background, perpetrator-victim involvement, and resolution of sexual harassment. This research used 20 eligible and relevant theses for the study of sexual harassment have been published from 2007 to 2022. The result showed that most cases occurred in urban areas, where the perpetrators are people known to the victim, such as family members, colleagues at work, neighbors, and playmates. Therefore, the role of non-governmental organizations and local governments is very helpful for victims to form coping strategies and report criminal cases. Sexual harassment needs to get more public attention by strengthening litigation and non-litigation needs, accompanied by the provision of sexuality education for parents, children, and the community.

## 1 Introduction

Sexual harassment is an intentional and purposeful act to intimidate, demean, humiliate or sexually assault the victim (Quick and McFadyen, 2017). It is also characterized by making unwelcome and inappropriate sexual remarks or physical advances in the workplace or other professional and social gatherings. The basic difference between sexual harassment and other behaviors is the willingness of the individuals involved, known as consent (Beres, 2014). In this disruption era, it is revealed to the public through media coverage and social-virtual networks, although many cases have ended without a clear resolution. According to Nurbayani et al. (2022a), victims of sexual harassment, including those in their immediate social environment, often regard the case as a disgrace. It sometimes triggers the emergence of post-traumatic stress disorder when victims not properly handled. In Indonesia, various sexual harassment has become a concern since the issue of women's protection and violence started in the parliament. The Indonesian Ministry of Education, Research and Technology has issued a minister regulation ‘Permendikbud’ Number 30/2021 concerning the Prevention and Handling of Sexual Violence in Higher Education (Adawiyah et al., 2022). The scope of policy which only revolves around educational institutions, is still not strong enough to protect potential victims, this is a signal that there are many cases of sexual harassment in the country.

The involvement of higher educational institutions, especially universities, in sexual harassment, can be viewed from an academic perspective (Clancy et al., 2020). Many faculties offer study programs (departments) and centers capable of researching this topic. This role further strengthens the knowledge and dedication of universities to the community and provides a tangible impact on the prevention of sexual harassment. In-depth studies are reflected in the final projects carried out by students under the lecturers supervision (Boud and Costley, 2007). These are experts in specific fields, and they are usually active in study groups and teach specialized courses. The final project also has to align with a concentration that students need to choose when they reach third academic year. It is common to find at least two concentration areas in any study program held at Indonesian campuses. The research output, final project, has received less attention from the public or fellow academics, even though the campus librarian had stored these documents online through a repository managed internally (Irawan et al., 2021). Several review studies on cases of sexual harassment only use databases from global indexers such as Copernicus, DOAJ, EBSCO, Scielo, Scopus, Web of Science, etc.

A few studies on sexual harassment adopted the systematic literature review (SLR) method. Karami et al. (2021) reported that the work environment was one of the popular themes raised. The most vulnerable are people with disabilities or those suffering from chronic diseases. Hunt et al. (2010) developed three models related to sexual harassment, such as primary, secondary, and intervention. However, McDonald (2012) focused on manuscripts that have been published over the past 30 years. It was reviewed that the management and organization, evaluation of evidence, and investigation of certain areas required further treatment. Victims of sexual harassment include adults, teenagers, and children. Popović (2018) stated that child sexual abuse studies are the basis of news media coverage, cases reported to the authorities, and media presentations.

Previous research seems to focus on articles published in journals or proceedings, despite the availability of library repositories provided by many campuses for the extraction of relevant information and its indexed by Google Scholar. They provide a repository to maximize the impact of students' final projects, avoid plagiarism, also encourage the reproducibility of scientific works (Farida et al., 2015; Azwar, 2017). Therefore, this research summarizes the studies on sexual harassment available in the online repository. It focused on undergraduate students' final projects, especially in the case studies, because the contents were more in-depth and spread across various areas. This study would be an inspirational and valuable input for policy development to overcome sexual harassment. Moreover, it also selects the appropriate treatment for both victims and perpetrators.

## 2 Research method

This study adopted a systematic literature review (SLR) which refers to the Preferred Reporting Items for Systematic Reviews and Meta-Analysis (PRISMA) (Valizadeh et al., 2021). SLR uses previous studies to answers research questions, and it differs from traditional literature reviews, it is more transparent, replicable, and scientific for theoretical development in specific fields (Puggina et al., 2018; Franco and Groesser, 2021). The protocol involved in this study has been systematically compiled and listed on OSF Registries (https://osf.io/5sm7g). OSF is a provider of protocol registration services for various studies, including for SLR studies and meta-analysis (Harrer et al., 2021).

### 2.1 Search strategy and eligibility criteria

The data was obtained from bachelor's theses at the online repositories of Islamic state higher education under the Indonesian Ministry of Religion Affairs, precisely at the campus classified as ‘Universitas Islam Negeri’ or UIN (The Islamic State University). Currently, 23 campuses are officially in this category, although this number tends to increase with the transformation movement of ‘Institut Agama Islam Negeri’ or IAIN (The Islamic State Institute) to UIN (Suharto and Khuriyah, 2014; Arifin, 2021). This transformation affects to scope of scientific activities, it becomes more flexible to study natural sciences, social sciences and humanities, and technology like secular campuses. Therefore, data sources were selected using several criteria, such as the establishment of UIN in 2017, which serves as an online repository that is open-access (full-text available), also has studied social sciences and humanities as reflected in the relevant faculty. This led to selection of 6 campuses from UIN Syarif Hidayatullah Jakarta, UIN Walisongo Semarang, UIN Sunan Ampel Surabaya, UIN Alauddin Makassar, UIN Ar-Raniry Aceh, and UIN Raden Fatah Palembang, as shown in Table 1. They provided open access, with some registered in the Directory of Open Access Repository (DOAR) (Priyanto, 2015). The search strategy used the keywords ‘pelecehan seksual’ (sexual harassment), ‘kekerasan seksual’ (sexual violence), and ‘kejahatan seksual’ (sexual crime).

**Table 1:** Library repository in the selected campus.

Campus	Repository Name	URL
UIN Syarif Hidayatullah Jakarta, Banten	Institutional Repository UIN Syarif Hidayatullah Jakarta	https://repository.uinjkt.ac.id/dspace/
UIN Walisongo Semarang, Central Java	Walisongo Institutional Repository	https://eprints.walisongo.ac.id/
UIN Sunan Ampel Surabaya, East Java	Digital Library UIN Sunan Ampel Surabaya	http://digilib.uinsby.ac.id/
UIN Alauddin Makassar, South Sulawesi	Repository UIN Alauddin Makassar	http://repositori.uin-alauddin.ac.id/
UIN Ar-Raniry Aceh, Nangroe Aceh Darussalam	Repository UIN Ar-Raniry Aceh	https://repository.ar-raniry.ac.id/
UIN Raden Fatah Palembang, South Sumatera	Repository UIN Raden Fatah Palembang	http://repository.radenfatah.ac.id

### 2.2 Selection process

Despite using these keywords, not all the titles were properly selected because the search field resulted 2533 thesis. Checking titles and abstracts to know the general content are needed. This step led to 35 theses because the titles and abstract sections contain a case study approach. These were carefully read to overview the research questions, its scope, and methodology. This research not differentiate theses in terms of systematic writing, because it usually adapts to the policies of each campus. Some faculties and study programs have a different thesis presentation, although it can be minor in the section or subsection (Paltridge, 2002; Paltridge and Starfield, 2007).

### 2.3 Data extraction

**Figure 1:**
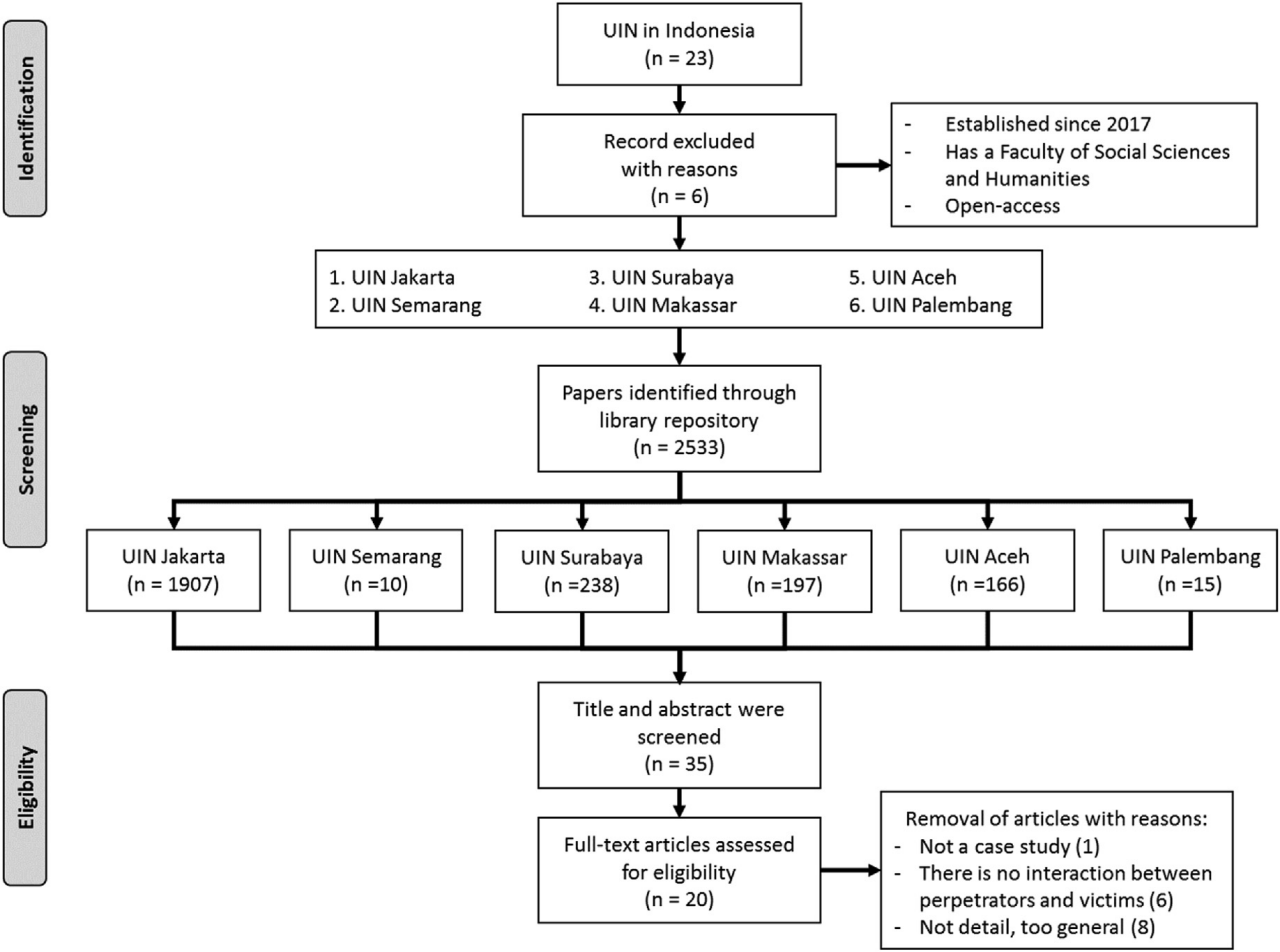
Selecting processes for the SLR analysis.

Assessment is the first step to ensure that a scientific work contains a relevant methodology to the research objectives (Snyder, 2019). In this context, these are suitable for SLR analyses, and it needs to contain an observational case study using quantitative or qualitative approaches. The full-text content sequentially starts with the introduction, theoretical studies, research methods, results and discussion, and finally, the concluding aspect, which is essential to provide a complete understanding of the writing purpose. Applying special criteria to sort out scientific works in the review process is necessary. The main criteria are must be a case study and the victims and perpetrators need to be involved. These stages found 20 theses are eligible for further analysis, and the details are shown in Figure 1. The authors carried out data extraction after initially agreeing to the criteria. Sexual harassment includes delinquency, physical violence, sexual assault, lewd conduct, and pedophilia (Nurbayani, 2021).

### 2.4 Quality assessment and data analysis

The main standard was set that all documents need to be open access from the cover to the appendix. At least five criteria were used to monitor the scientific work for SLR analysis, 1) undergraduate thesis and is guided by at least one supervisor; 2) origin of the relevant study program and faculty; 3) presenting case studies; 4) serving the background information on locations, perpetrators, and victims who contain their interaction; 5) presenting case's resolution. Two investigators also performed this assessment to evaluate the feasibility of theses as study material and discuss it with the authors. Quality assessment involving other parties is an extra effort because the involvement of more experts is better than one (Priharsari, 2022). Data from these were further processed using the content analysis method, which is useful for obtaining in-depth written information (Khirfan et al., 2020a, Khirfan et al., 2020b; Lee et al., 2021). A qualitative approach provides various flexible interpretations, builds interconnections, emphasizes problems, and discerns gaps (Khirfan et al., 2020a, Khirfan et al., 2020b).

## 3 Results

Most of the research on sexual harassment (80%) are from Java Island, spreading across UIN Jakarta, UIN Semarang, and UIN Surabaya with nine, four, and three theses, respectively. Those from UIN Palembang and UIN Makassar published in the past five years – after 2017 were included (Table 2). UIN Aceh did not fit the criteria, therefore the works were excluded from this analysis. The past eligible theses used to discuss this issue were obtained from UIN Jakarta, where sexual harassment has been discussed since 2007 by Karyanti. Meanwhile, Sunti, 2022 carried out a study on verbal sexual harassment in Surabaya. The difference in distribution proves that state universities in Sumatra, Kalimantan, and Sulawesi were formerly branches of campuses on Java (Esha, 2018). From the snowball search for references, no other eligible theses were discovered.

**Table 2:** Summary of bachelor theses identity.

Affiliation		Author
UIN Jakarta	Faculty of Da'wah and Communication Studies	Huwaidah (2011), Al-Ashzim (2018), Fadilah (2018), Purba (2020) and Azami (2021)
	Faculty of Social and Political Sciences	Permatasari, 2018 and Hanifah (2021)
	Faculty of Sharia and Law	Alifia (2021)
	Faculty of Psychology	Karyanti (2007)
UIN Semarang	Faculty of Da'wah and Communication Studies	Nafisah (2015), Anggraini (2017), Soraya (2018) and Hikmah (2020)
UIN Surabaya	Faculty of Da'wah and Communication Studies	Ilkafah, 2014 and Zakaria (2014)
	Faculty of Theology and Philosophy	Sunti, 2022
UIN Palembang	Faculty of Da'wah and Communication Studies	Dinda (2018), Pauziah (2018) and Fatih (2019)
UIN Makassar	Faculty of Medicine and Health Sciences	Ramadhani (2018)

### 3.1 Background of sexual harassment cases

Sexual harassment can happen anywhere, especially when the victim is inattentive and creates obscene opportunities for the perpetrator. This heinous act is usually carried out in urban areas. Therefore, these communities are vulnerable to sexual threat. Several studies have been carried out in rural areas by Al-Ashzim (2018), Permatasari, 2018, and Hikmah (2020). Permatasari, 2018 carried out unique research, where four cases occurred in urban areas, and one was raised in rural communities. In addition, sexual harassment also existed in private and public spaces such as mass transportation facilities, social-public amenities, educational institutions, and roads (Pauziah, 2018; Hanifah, 2021; Sunti, 2022). Many cases occurred in these places carefully mapped out by the perpetrator, and the victims fell into their traps.

Based on detailed observations, narrow and quiet facilities such as bedrooms, boarding rooms, toilets, and workspaces are potential places that require extra vigilance against sexual harassment (Ilkafah, 2014; Dinda, 2018; Fadilah, 2018). These known as ideal locations, especially if the perpetrator has detailed insight into the area. Empty houses that had been abandoned for a long time or temporarily by its occupants is usually the scene place. The victim may have only just realized incidentally and cannot take preventive measures. Nevertheless, a known environment does not guarantee one's safety because several cases occurred in the victims' homes (Zakaria, 2014; Soraya, 2018). Details about the social settings and locations where this heinous act occurred are shown in Table 3.

**Table 3:** Cases background in the eligible bachelor's theses.

Event setting	Place scene
Urban	Houses of friends or relatives (Karyanti, 2007); play environment around the house, house (Huwaidah, 2011); boarding house (Ilkafah, 2014); house (Zakaria, 2014); the perpetrator's house, the victim's house, lodging (Nafisah, 2015); house rooms, hotels (Anggraini, 2017); the perpetrator's house (Dinda, 2018); work space, room, quiet place, abandoned school building, bathroom at a friend's house (Fadilah, 2018); next to the shop (Pauziah, 2018); the victim's house, the perpetrator's house (Ramadhani, 2018); the victim's house, tourist attractions, the perpetrator's house (Soraya, 2018); houses, public transportation, roads (Fatih, 2019); houses, alleys near the victim's house, lodging (Purba, 2020); houses, school toilets, boarding rooms (Alifia, 2021); the perpetrator's house (Azami, 2021); KRL Commuter Line (Hanifah, 2021); around Depok train station; deserted/empty train carriages, academic environment, roads, public transportation (Sunti, 2022)
Rural	House (Al-Ashzim, 2018); house, neighbor's house (Hikmah, 2020)
Periurban, suburban	The perpetrators' houses or their relatives, the victim's house, and place of work (factory) (Permatasari, 2018)

### 3.2 Involvement between perpetrators and victims

Perpetrators and victims of sexual harassment belong to various age groups, and they are usually children (3–12 years), teenagers (13–18 years), and adults (19–64 years old). The interaction between them is not only an attraction due to gender differences, but it also includes homosexuals and fetish, for example, the urge to have sex with children (Huwaidah, 2011; Nafisah, 2015; Anggraini, 2017; Dinda, 2018; Pauziah, 2018; Hikmah, 2020; Purba, 2020; Azami, 2021). In several eligible theses, the majority of the incidents only occurred between the victim and the perpetrator. However, in some circumstances, the sexual predators tend to appear where a perpetrator has harassed many victims, besides, this is usually related to pedophilia (Alifia, 2021). On the other hand, the theses that discussed the existence of victims harassed by several perpetrators in a heterosexual context was also discovered (Zakaria, 2014). From all case studies that have been reviewed, there are approximately 48 victims and 63 perpetrators. The majority of perpetrators are adults (approximately 44 persons), then followed by teenagers (approximately 17 persons) and children (two person). Meanwhile, in terms of victims, they are usually children (12), teenagers (27), and adults (8).

Sexual harassment cases are real and arise from the perpetrator's awareness. It is usually planned, and the potential victim is lured to the scene. In certain cases, especially in public places, the perpetrator is not known to the victim. They are cigarette traders, buskers, and fellow passengers on public transportation (Karyanti, 2007). From the eligible theses, perpetrators are mainly motivated to satisfy their depraved lust (Table 4). They also dare to engage in this heinous act because they know or are close to the victim (Permatasari, 2018; Azami, 2021). Social relationships can take the victim off guard. It is beneficial to the perpetrator, they get the absolute trust from victims and its community (Pauziah, 2018; Hanifah, 2021; Sunti, 2022). Perpetrators of sexual harassment are not only friends, co-workers, girlfriends, or lovers, neighbors, teachers, and strangers often met outside the home (Alifia, 2021). However, it also involves intimate relations, such as fathers, uncles, grandfathers, and younger cousins. The occurrence of sexual harassment among family members can even involve toddlers (Hikmah, 2020). The perpetrator engages in sexual intercourse on the pretext of fulfilling biological needs, which has been prolonged.

**Table 4:** The motivation of the perpetrators and their relationship with the victims.

Perpetrators motivation	Satisfy sexual desire (Karyanti, 2007; Huwaidah, 2011; Nafisah, 2015; Anggraini, 2017; Ilkafah, 2014; Dinda, 2018; Pauziah, 2018; Ramadhani, 2018; Soraya, 2018; Fatih, 2019; Hikmah, 2020; Purba, 2020; Hanifah, 2021)
	There is a chance (Dinda, 2018; Pauziah, 2018; Fatih, 2019)
	Curious (just experimenting) (Ramadhani, 2018; Permatasari, 2018)
	Want to feel pleasure; ensure that the relationship between the two parties continues to last (Permatasari, 2018)
	Long time no sex with wife, sexual fantasies because they often watch pornographic videos, revenge that leads to a cycle of sexual harassment (Huwaidah, 2011)
	Feel close and feel free to do whatever (Azami, 2021)
	Sexual disorders (exhibitionism) (Karyanti, 2007)
	Using victims with disabilities (disability) (Soraya, 2018)
	Victims often wear tight clothes with open tops (Ilkafah, 2014)
	Just a fad (joking) (Sunti, 2022)
Involvement between perpetrators and victims	Family (Soraya, 2018), biological father (Zakaria, 2014; Anggraini, 2017; Al-Ashzim, 2018; Hikmah, 2020; Alifia, 2021), step father (Nafisah, 2015; Ramadhani, 2018; Hikmah, 2020); uncle (Huwaidah, 2011; Fatih, 2019), grandfather (Purba, 2020), younger cousin (Ramadhani, 2018)
	Unmarried couple (boyfriend or girlfriend) (Ilkafah, 2014; Nafisah, 2015; Anggraini, 2017; Fadilah, 2018; Soraya, 2018; Ramadhani, 2018; Permatasari, 2018; Purba, 2020)
	Neighbor (Huwaidah, 2011; Anggraini, 2017; Al-Ashzim, 2018; Dinda, 2018; Fadilah, 2018; Ramadhani, 2018; Soraya, 2018; Hikmah, 2020; Alifia, 2021; Azami, 2021)
	Friend (Karyanti, 2007; Fadilah, 2018; Permatasari, 2018; Sunti, 2022)
	Colleagues – boss, employer, fellow buskers (Karyanti, 2007; Nafisah, 2015; Fadilah, 2018)
	Employees or staff in educational institutions (teachers, security guards) (Fadilah, 2018; Alifia, 2021)
	Other people – parents' colleagues (Zakaria, 2014), friends of the perpetrator (Nafisah, 2015), dad with his neighbor (Anggraini, 2017), son of the owner of the boarding house (Alifia, 2021)
	New people are known, including from social media (Al-Ashzim, 2018; Soraya, 2018)
	Stranger (Karyanti, 2007; Pauziah, 2018; Fatih, 2019; Hanifah, 2021; Sunti, 2022)

A lack of parental caring is mostly causes sexual harassment involving children or adolescents. It can also be driven by the state of playing group, Internet content that encourages sexuality, and social media (Huwaidah, 2011; Permatasari, 2018; Soraya, 2018; Purba, 2020). In most cases, there is a lack of sexuality education among the parties involved (Hanifah, 2021), and this is caused by the victim's carelessness or silence (Fadilah, 2018), as well as psychiatric disorders and past trauma (Alifia, 2021). In several cases, perpetrators often take advantage of the victims' unfavorable situations, such as economic difficulties, disability, poverty, difficulty in accessing jobs, broken homes, and natural disasters (Karyanti, 2007; Anggraini, 2017). Based on socio-economic terms, they are powerless and unable to fight back due to male superiority (Nafisah, 2015; Fatih, 2019). This unequal relationship is one of the factors causing various cases of sexual harassment to be difficult to uncover, and even tend to be completed without any meaningful legal action. This detrimental situation threatens the victims' lives, and they experience all forms of sexual harassment, from verbal, touching body parts, forceful to touching or seeing the perpetrator's vital organs, exhibitionism, coercion, and persuasion to have oral, vaginal, or anal sex (Table 5).

**Table 5:** Sexual harassment of the victim.

The act and intensity of sexual harassment	Reference
Having sex (frequently), some victims become pregnant	Fadilah (2018); Permatasari, 2018; Ramadhani (2018); Soraya (2018); Hikmah (2020); Purba (2020); Alifia (2021)
Rape	Huwaidah (2011); Ilkafah, 2014; Anggraini (2017); Fadilah (2018); Soraya (2018); Purba (2020)
Raped by more than one perpetrator at the same time	Nafisah (2015); Fadilah (2018);
Teasing and saying vulgar (verbal) words, including catcalling	Karyanti (2007); Fadilah (2018); Sunti, 2022
Groping the whole body or sensitive parts (foundlings) such as the waist, buttocks, breasts, or vagina	Karyanti (2007); Nafisah (2015); Anggraini (2017); Fadilah (2018); Ramadhani (2018); Fatih (2019); Azami (2021); Hanifah (2021)
Sniffing the head, kissing the face	Karyanti (2007); Soraya (2018); Hanifah (2021)
Sticking the genitals (penis), holding the buttocks	Hanifah (2021); Alifia, 2021
Inserting finger or blunt objects into the vagina, thereby causing injury	Anggraini (2017); Huwaidah (2011); Alifia (2021)
Sodomy (anal)	Al-Ashzim (2018); Soraya (2018); Hikmah (2020); Alifia (2021)
Asking the victim to sit on the perpetrator's lap	Fadilah (2018)
Having violent sex	Pauziah (2018); Hikmah (2020); Alifia (2021)
Inviting and forcing the victims to watch porn	Karyanti (2007)
Hugging the victim without consent	Karyanti (2007); Dinda (2018)
Exhibitionism	Karyanti (2007); Fatih (2019)
Forced to suck the penis	Anggraini (2017)
Taught to play (hold) with the perpetrator's penis and ass	Anggraini (2017)
Forceful sex	Zakaria (2014)

Victims experience various forms of sexual harassment because of their helplessness, and some are threatened. Soraya (2018) reported a case where the victim was forced to kiss the perpetrator, also the act was recorded and shared on social media (Facebook). Perpetrators make tempting promises, such as asking for marriage, shouldering every pregnancy responsibility, persuasion, and offering interesting gifts (Fadilah, 2018; Ramadhani, 2018; Purba, 2020; Alifia, 2021; Azami, 2021). Victims can also be made unconscious by either making them drunk or falling asleep (Permatasari, 2018). Threats to be tainted, physical violence, and extortion were also reported (Karyanti, 2007; Soraya, 2018). Some victims receive death threats if they do not comply with the perpetrator's wishes or report the case (Zakaria, 2014; Nafisah, 2015; Anggraini, 2017; Dinda, 2018; Pauziah, 2018; Hikmah, 2020). Perpetrators are not limited to harassing, they also insult their victims (Ilkafah, 2014). Some victims tend to feel frozen because, in most circumstances, they are too shocked to speak up (Fatih, 2019). This heinous act leads to the emergence of fear, regret, shame, sadness, and depression. The victims never anticipated it, sexual harassment making them to live a normal life again (Table 6).

**Table 6:** The impact of sexual harassment on victims.

Physical impact	• Injuries on body parts (Ilkafah, 2014) • Wounds on the genitals (Purba, 2020) • There is discomfort around the genitals (Anggraini, 2017) • Infected with infectious disease (Anggraini, 2017) • Pregnant (Anggraini, 2017; Alifia, 2021) • Pain when urinating (Alifia, 2021) • Get sick easily (Alifia, 2021) • Unstable heartbeat (palpitations easily) (Pauziah, 2018) • Frequent urination (Pauziah, 2018)
Socio-psychological impact	• Fearful (Karyanti, 2007; Nafisah, 2015; Al-Ashzim, 2018; Dinda, 2018; Fadilah, 2018; Permatasari, 2018; Ramadhani, 2018; Soraya, 2018; Azami, 2021; Hanifah, 2021) • Shame (Huwaidah, 2011; Anggraini, 2017; Ramadhani, 2018; Hanifah, 2021) • Feeling guilty and regret (Karyanti, 2007; Fadilah, 2018; Ramadhani, 2018; Permatasari, 2018; Sunti, 2022) • Trauma (Karyanti, 2007; Huwaidah, 2011; Anggraini, 2017; Al-Ashzim, 2018; Dinda, 2018; Soraya, 2018; Fatih, 2019; Hikmah 2020) • Quiet, moody, often aloof and likes to daydream (Huwaidah, 2011; Dinda, 2018; Soraya, 2018; Hikmah 2020; Alifia, 2021) • Feeling depressed and hurt (Huwaidah, 2011; Ramadhani, 2018) • Difficulty socializing or withdrawing from surroundings (Anggraini, 2017; Al-Ashzim, 2018; Dinda, 2018; Fatih, 2019; Hikmah, 2020) • Suspicion increases, limits relationships, very selective in choosing colleagues, difficult to trust others or unknown people, including adults (Karyanti, 2007; Anggraini, 2017; Fadilah, 2018; Pauziah, 2018; Ramadhani, 2018; Fatih, 2019; Sunti, 2022) • Sleep disorders and nightmares (Anggraini, 2017; Pauziah, 2018; Soraya, 2018; Fatih, 2019; Purba, 2020) • Unstable emotions, easily sad, cries, and screams often (Karyanti, 2007; Fadilah, 2018; Pauziah, 2018; Ramadhani, 2018; Soraya, 2018; Fatih, 2019; Purba, 2020; Alifia, 2021; Azami, 2021; Sunti, 2022) • Difficulty concentrating, on work or other activities (Karyanti, 2007; Pauziah, 2018; Soraya, 2018; Fatih, 2019) • Helpless, lethargic, and weak (Anggraini, 2017; Fadilah, 2018; Soraya, 2018) • Stop or disruption of formal education activities (Fadilah, 2018; Soraya, 2018; Hikmah, 2020) • Depression, frustration, and hopelessness (Zakaria, 2014; Fadilah, 2018; Ramadhani, 2018) • Anxious and restless (Anggraini, 2017; Nafisah, 2015; Pauziah, 2018; Soraya, 2018; Fatih, 2019) • Likes to hit hard objects (Soraya, 2018) • Loss of confidence (Anggraini, 2017) • Eating disorders (Anggraini, 2017) • Feeling disgusted or sordid (Ramadhani, 2018)

### 3.3 Cases resolution

Sexual harassment can be tackled if the victim is willing to disclose it to others, as reported by the majority of the eligible theses. In certain cases, the closest people need to be extremely sensitive because not all victims are willing to speak, however it can be recognized from changes in attitudes, behavior, even a number of injuries on the person's limbs (Dinda, 2018; Soraya, 2018; Purba, 2020; Azami, 2021). They consist of family (parents, brothers, uncles or aunts, grandparents), neighbors, and teachers (Zakaria, 2014; Alifia, 2021). Closest people, especially parents, can continue to investigate these changes, including the victim's socialization pattern with the suspected person (Fadilah, 2018; Pauziah, 2018). Other people is important because they can facilitate the victims, to take appropriate actions such as getting the perpetrators arrested (Permatasari, 2018). Disclosure aids them to get support from local governments, police, schools, social workers, and non-governmental organizations (Al-Ashzim, 2018). Some victims dared to report this case independently to the mediator or the authorities (Ilkafah, 2014; Nafisah, 2015). Non-governmental organizations are preferred by victims as mediators. The cases usually get a referral and can be handled by the police, also the victims tend to be stable after the incident. Although, certain victims prefer to keep this case to themselves, unless there are some parties who try to open the veil (Fatih, 2019).

The ability to handle sexual harassment by the victims and those around them is caused by several motivations, including 1) the family finds it difficult to accept such act, feels hurt, and acts outrageous (Ilkafah, 2014; Nafisah, 2015; Anggraini, 2017; Fadilah, 2018; Soraya, 2018; Permatasari, 2018; Alifia, 2021; Azami, 2021); 2) reports the incident because it has been troubling them (Fadilah, 2018; Permatasari, 2018); 3) to receive special assistance and treatment (Al-Ashzim, 2018; Ramadhani, 2018); 4) the victim was physically injured, or even got pregnant (Purba, 2020; Alifia, 2021); 5) feeling helpless (Nafisah, 2015); 6) loss of valuables that were taken away by the perpetrators (Anggraini, 2017). Sexual harassment always leaves an imprint on the victim, although its extent differs depending on personal situation, post-case handling, and the level of trauma. Based on these studies, there are many trauma handling and coping strategies of the victims as shown in Table 7. Unfortunately, only four theses stated that these perpetrators are legally processed by the police (Anggraini 2017; Soraya 2018; Permatasari, 2018; Purba 2020).

**Table 7:** Trauma treatment and coping strategy for victims of sexual harassment.

Trauma treatment	• Providing support groups (seminars and discussions) (Huwaidah, 2011; Hikmah, 2020) • Story telling activities about Islam (Huwaidah, 2011) • Directive services (Huwaidah, 2011) • Playing method (Huwaidah, 2011; Hikmah, 2020) • Relocation of residence and school (Al-Ashzim, 2018; Purba, 2020) • Sexual orientation recovery (Al-Ashzim, 2018) • Remembrance and mourning (Fadilah, 2018) • Medical support (Nafisah, 2015; Soraya, 2018; Hikmah, 2020) • Drawing method (Al-Ashzim, 2018; Soraya, 2018) • Method of suppressing emotions (Soraya, 2018) • Motivational giving (Soraya, 2018) • Facilitated shelter (safe house) (Nafisah, 2015; Anggraini, 2017) • Family therapy (Hikmah, 2020) • Human validation process model (Hikmah, 2020) • Repatriation and social reintegration (Anggraini, 2017) • Reality therapy (Ilkafah, 2014) • Strengthen resilience (emotional regulation, optimism, self-efficacy, causal analysis, impulse control, empathy, and reaching out) (Zakaria, 2014) • Psychodrama technique (Pauziah, 2018) •Islamic counseling (Fatih, 2019)
Coping strategy	• Asking for patience and gratitude (Huwaidah, 2011) • Divert trauma through various other activities (Hanifah, 2021) • Individual empowerment (Al-Ashzim, 2018)

## 4 Discussion

Eligible theses that discussed sexual harassment were obtained from social and humanities study programs (departments), mainly came from Islamic Guidance and Counseling (45%), Social Welfare (20%), Psychology (10), Sociology (10%), Public Health (5%), Family Law (5%), as well as Islamic Faith and Philosophy (5%). This situation illustrates that the study is multidisciplinary, interdisciplinary, and transdisciplinary. Even though in the academic context, these studies have not yet received full support due to the clear separation of scientific fields through formal regulations implemented by the Indonesian Ministry of Education, Research and Technology (Fitri et al., 2020). Handling sexual harassment requires the adoption of approaches from various related and allied scientific fields, both in pre and post-events against victims, the surrounding environment, also the perpetrators. It is also important to change perspectives and to be more concerned about the victims, because patriarchal societies tend to blame them, they provided the opportunity for perpetrators to commit these heinous acts (O'Donohue et al., 1998). A study carried out by Hanifah (2021), stated that politeness, and loose clothing also does not guarantee one's freedom from sexual harassment. The factor within the perpetrator is the main control this phenomenon (Shakeshaft, 2004; Greathouse et al., 2015). Irrespective of this, creating a safe environment is a shared responsibility of all societal elements and needs to be supported by tiered formal regulations (Wurtele, 1987; Barker, 2017; Susiana, 2021). The formation of a task force at the school level, even hamlet in the context of Indonesia's territory, can be the key control.

The majority of sexual harassment cases were not reported because of the victim's disadvantaged position and the perpetuator's connections, family, threats, education background, also socio-economic aspects (Ward and Rodger, 2018; Aguilar and Baek, 2020; Wood et al., 2021). According to Huwaidah (2011) there are numerous obstacles related to the legal processing of sexual harassment cases, especially if the perpetuators were underage and the victim an adult. Most cases are only reported to non-governmental organizations or local government institutions because victims are reluctant to report it to the police for various reasons, such as lack of evidence, shame, and financial restraints (Dinda, 2018; Pauziah, 2018; Hanifah, 2021). Perpetrators need to be punished and treated as criminals, and made not to commit this heinous act again before resocialization. The lack of strong evidence to drag perpetrators clearly demands the attention of academics and researchers to develop a robust assessment instrument capable of revealing latent aspects of both parties (Erwinda et al., 2020). Sexual harassment should not be a biased concept due to a change from the consent relationship between the two parties to the criminalization process without a detailed investigation, including the case of lovers or married couples (Refinetti, 2018).

The victim-oriented resolution really requires sensitive people around, especially parents. They play a significant role in the case of children and adolescents. Victims of sexual violence often exhibit somatic complaints without any organic basis. According to Kendall-Tackett et al. (1993), and Sweeting et al. (2022), both men and adults experience trauma, increasing competence among parents regarding sexuality education plays a role, although this has challenges such as educational background and society. Parents also need to teach sexuality education to their children, they capable to build intimate and open communication (Nurbayani et al., 2022b). This applies to all sexes and not only a particular gender, considering that cases of sexual harassment can happen to both men and women, thereby enabling them to have self-control (Parkes et al., 2016). Sexual harassment also needs to be understood as an event that can happen anywhere, including places that were previously considered safe by victims and their parents – homes, educational institutions, workplaces, and playing environments, therefore one always need to be at alert attitude (Hill and Kearl, 2011; Bondestam and Lundqvist, 2020; Adams et al., 2021). It is equally important, to provide trauma care for victims. This need to be able to stop imitating behavior that is not only carried out by psychiatrists, but also by parents and families who can interact more intensively. Victims also need to continuously receive attention in the form of litigation and non-litigation assistance to get the ideal coping strategy.

## 5 Conclusion

The SLR focuses on sexual violence by utilizing undergraduate theses obtained from a library repository at the Islamic campuses (UIN) spread across Indonesia. The criteria for determining the theses were clearly defined. Therefore, the discussion remains focused and detailed. There are 23 UINs in Indonesia, but only six are open-access repository. However, in the end only 20 theses met all the criteria. The assessment process refers to certain criteria such as theses originating from the social sciences and humanities which present case studies, including background, setting of the incident in both urban or rural environments, as well as specific places, motivations, and interactions between victims and perpetrators. In addition, the theses contain efforts and strategies for solving these cases. The majority of the studies on sexual harassment were obtained from Java, such as UIN Jakarta (nine theses), UIN Semarang (four theses), and UIN Surabaya (three theses).

Sexual harassment can occur anywhere, however it is rampant in urban environments and sometimes executed in a quiet place far from social activities, such as roads, public amenities, and empty house. Furthermore, this crime can be committed by close family members, lovers, neighbor, or even strangers. Most of the victims are children and adolescent, while the perpetrators are generally adults, some teenagers, and a few children. Various motivations that trigger these actions include wanting to satisfy lust, opportunity, fad or trial and error. Sexual harassment is triggered by lack of supervision from parents, poverty, ease of internet access and other external conditions. It causes adverse impact on the victim's physical and psychological conditions who hardly win such cases in court. Awareness is needed from various parties to resolve sexual crimes that are currently common. Presently a number of non-governmental and local government institutions seek to provide trauma management and appropriate coping strategies.

## Declarations

### Author contribution statement

All authors listed have significantly contributed to the development and the writing of this article.

### Declaration of interests

The authors declare no conflict of interest.

### Data availability statement

Data will be made available on request.

### Funding statement

This work was supported by the Faculty of Social Sciences Education (FPIPS) and Universitas Pendidikan Indonesia in 2022 through “program publikasi ilmiah bereputasi” on behalf of the first author.

### Additional information

No additional information is available for this paper.
